# SLC37A1 and SLC37A2 Are Phosphate-Linked, Glucose-6-Phosphate Antiporters

**DOI:** 10.1371/journal.pone.0023157

**Published:** 2011-09-20

**Authors:** Chi-Jiunn Pan, Shih-Yin Chen, Hyun Sik Jun, Su Ru Lin, Brian C. Mansfield, Janice Y. Chou

**Affiliations:** Section on Cellular Differentiation, Program in Developmental Endocrinology and Genetics, Eunice Kennedy Shriver National Institute of Child Health and Human Development, National Institutes of Health, Bethesda, Maryland, United States of America; Technion-Israel Institute of Technology Haifa 32000 Israel, Israel

## Abstract

Blood glucose homeostasis between meals depends upon production of glucose within the endoplasmic reticulum (ER) of the liver and kidney by hydrolysis of glucose-6-phosphate (G6P) into glucose and phosphate (P_i_). This reaction depends on coupling the G6P transporter (G6PT) with glucose-6-phosphatase-α (G6Pase-α). Only one G6PT, also known as SLC37A4, has been characterized, and it acts as a P_i_-linked G6P antiporter. The other three SLC37 family members, predicted to be sugar-phosphate:P_i_ exchangers, have not been characterized functionally. Using reconstituted proteoliposomes, we examine the antiporter activity of the other SLC37 members along with their ability to couple with G6Pase-α. G6PT- and mock-proteoliposomes are used as positive and negative controls, respectively. We show that SLC37A1 and SLC37A2 are ER-associated, P_i_-linked antiporters, that can transport G6P. Unlike G6PT, neither is sensitive to chlorogenic acid, a competitive inhibitor of physiological ER G6P transport, and neither couples to G6Pase-α. We conclude that three of the four SLC37 family members are functional sugar-phosphate antiporters. However, only G6PT/SLC37A4 matches the characteristics of the physiological ER G6P transporter, suggesting the other SLC37 proteins have roles independent of blood glucose homeostasis.

## Introduction

The glucose-6-phosphate transporter (G6PT, also known as SLC37A4) is localized in the endoplasmic reticulum (ER) membrane [1], [2], where it acts as a phosphate (P_i_)-linked, glucose-6-phosphate (G6P) antiporter [3]. The primary function of G6PT is to translocate G6P from the cytoplasm into the lumen of the ER for hydrolysis into glucose and P_i_. The hydrolysis is catalyzed by a second ER membrane protein, glucose-6-phosphatase-α (G6Pase-α or G6PC), which has its active site localized within the lumen of the ER [4]. Together, the G6PT/G6Pase-α complex maintains interprandial glucose homeostasis. Deficiencies in G6PT cause glycogen storage disease type Ib (GSD-Ib) and deficiencies in G6Pase-α cause GSD-Ia [5], [6]. Both disorders manifest a metabolic phenotype of impaired glucose homeostasis [5], [6]. Notably, the G6PT activity is sensitive to inhibition by chlorogenic acid (CHA) [7]. Studies have also shown that G6PT-mediated microsomal G6P uptake activity requires an active G6Pase-α [8]. Hepatic microsomes prepared from G6Pase-α-deficient (GSD-Ia) mice, which still possess an intact G6PT, exhibit markedly lower G6P uptake activity compared to wild type hepatic microsomes [8]. However, this can be reversed if G6Pase-α activity is restored in the GSD-Ia mouse livers via gene transfer studies [9]. Based on these characteristics, functional assays to detect physiologically important G6PT activity have been developed [10]–[12].

The solute carrier 37 (SLC37) family consists of 4 proteins, SLC37A1 [13]–[15], SLC37A2 [15]–[17], SLC37A3 [15] and G6PT/SLC37A4 [5], [6], [15]. With the exception of G6PT, which was shown to function as a P_i_-linked G6P antiporter [3], none of the other SLC37 proteins has been functionally characterized. Their grouping into the SLC37 family is based on sequence homology to bacterial organo-phosphate:P_i_ exchangers [15]. SLC37A1 was cloned based on its similarity to the glycerol-3-phosphate transporter [13]. While SLC37A2 and SLC37A3 have no known substrates preferences, their similarity to G6PT/SLC37A4 suggests G6P might be a good candidate. In further support of this hypothesis, Leuzzi et al. [18] have shown that there is more than one G6P transport activity present in microsomes prepared from human fibroblasts and HeLa cells, and that these can be differentiated by their sensitivity to CHA. Only G6PT/SLC37A4 has been linked to a genetic disorder [5], [6]. SLC37A1 was not found to be mutated in patients with glycerol kinase deficiency, and despite mapping to the critical region of the autosomal recessive deafness locus, DFNB10 on chromosome 21q22.3, mutation analyses excluded it as the gene for DFNB10 [13]. Therefore, beyond the structural similarity, there is a need to understand the functional similarities of these proteins to gain insight into their biological roles. The first aims of this study were to establish whether the other SLC37 family members, SLC37A1, SLC37A2, and SLC37A3 are associated with the ER membrane and whether they function as P_i_-linked G6P antiporters. We then proposed to examine those ER-associated G6P transporters to determine if they can form a functional complex with G6Pase-α capable of hydrolyzing G6P to glucose; and if their activity is sensitivity to CHA. We show that both SLC37A1 and SLC37A2 meet the first criteria, however, unlike G6PT, neither is sensitive to inhibition by CHA and neither can couple functionally to G6Pase-α. In contrast, SLC37A3 is not a G6P transporter. Taken together these findings suggest that only SCL37A4 contributes to interprandial blood glucose homeostasis.

## Materials and Methods

### Construction of recombinant adenoviruses

The SLC37A1, SLC37A2 or SLC37A3 constructs were generated by PCR using the respective cDNA clones (Invitrogen, Carlsbad, CA) as templates. A sequence encoding the eight-amino-acid Flag marker peptide, DYKDDDDK (Scientific Imaging Systems, Eastman Kodak, CT) was added at the 5′ end of each construct, and the resulting DNA cloned into the pAdlox vector [19]. The nucleotide sequence in all constructs was verified by DNA sequencing. The recombinant adenovirus (Ad) was generated by the Cre-lox recombination system [19], resulting in Ad-SLC37A1-5Flag, Ad-SLC37A2-5Flag, and Ad-SLC37A3-5Flag. Recombinant Ad, Ad-G6PT, Ad-G6PT-5Flag, Ad-G6Pase-α, and Ad-G6Pase-β have been described previously [4], [12], [20]. Each recombinant virus was plaque purified and amplified to produce viral stocks with titers of approximately 1 to 3×10^10^ plaque forming unit (PFU) per ml.

### Expression in COS-1 cells and microsomal G6P uptake assay

COS-1 cells were plated in 150-cm^2^ flasks and grown at 37°C in HEPES-buffered Dulbecco's modified minimal essential medium supplemented with 4% fetal bovine serum. The multiplicity of infection (MOI) for Ad-G6PT, Ad-SLC37A1-5Flag, Ad-SLC37A2-5Flag, or Ad-SLC37A3-5Flag was 50 PFU/cell and MOI for Ad-G6Pase-α or Ad-G6Pase-β was 25 PFU/cell. Mock infected COS-1 cells were used as controls. After incubation at 37°C for 24 h, the infected cultures were used for microsome isolation, proteoliposome reconstitution, and Western-blot analysis.

Microsomal G6P uptake measurements were performed essentially as described previously [10]–[12]. Microsomes (40 µg) were incubated in a reaction mixture (100 µl) containing 50 mM sodium cacodylate buffer, pH 6.5, 250 mM sucrose, and 0.2 mM [U-^14^C]G6P (50 µCi/µmol, American Radiolabeled Chemicals, St Louis, MO). The reaction was stopped at the appropriate time by filtering through a nitrocellulose membrane (0.45 µm, Millipore Co., Billerica, MA), then washing with an ice-cold solution containing 50 mM Tris-HCl, pH 7.4 and 250 mM sucrose. The dried filters were counted in a liquid scintillation counter.

### Immunofluorescence microscopy

COS-1 cells (5×10^4^/chamber) in a 2-chambered coverglass (Nalge Nunc International Co, Naperville, IL) were infected with Ad-SLC37A1-5Flag, Ad-SLC37A2-5Flag, Ad-SLC37A3-5Flag, or Ad-G6PT-5Flag at MOI of 0.1 PFU/cell. After incubation at 37°C for 1 day, the infused cells were fixed for 10 min at 25°C in 3.7% formaldehyde in PBS, then permeabilized with 0.2% (v/v) Triton X-100 for 15 min at 25°C. After blocking with 5% (v/v) goat serum in PBS for 1 h at 25°C, the cells were incubated overnight at 4°C in PBS containing 5% goat serum with either a mouse monoclonal antibody against the Flag epitope (Sigma, St. Louis, MO), or a rabbit polyclonal antibody against calreticulin (Santa Cruz Biotechnology, Santa Cruz, CA). After washes with PBS, the cells were incubated for 1 h at 25°C in the dark, in PBS containing 5% goat serum with either goat anti-mouse or anti-rabbit antibody conjugated with Alexa Fluor 555 or 488 (Invitrogen, Carlsbad, CA) respectively. After washing with PBS, the cells were mounted with an anti-fade, water-based mounting medium containing DAPI (Vector Lab, Burlingame, GA), observed with an Axioskop 2 plus fluorescence microscope (Carl Zeiss, Thornwood, NY) and the images digitized using AxioVision 4.3 (Carl Zeiss). Co-localization of green fluorescent calreticulin and red fluorescent SLC37 protein results in a yellow fluorescence.

### Solubilization and reconstitution of membrane proteins

Solubilization of microsomal membrane proteins was performed as described previously [21]–[23]. Membrane proteins were solubilized on ice by mixing 2 mg of microsomes in 1 ml of a solution containing: 20 mM Tris-HCl, pH 7.5; 20% glycerol; 1.25% (w/v) *n*-octyl-β-D-glucopyranoside octylglucoside (Calbiochem, San Diego, CA); 2 mM DTT; 1% aprotinin; 1 mM AEBSF; 2 µg/ml pepstatin A; 2 µg/ml leupeptin (all inhibitors from Roche Diagnostics, Indianapolis, IN); and 0.4% (w/v) lipid mixture. The lipid mixture consisted of: *E coli* polar lipid extract, L-α phosphotidylcholine, L-α phosphotidylserine, and cholesterol combined in a ratio of 60∶17.5∶10∶12.5 w/w (all from Avanti Polar lipids, Inc, Alabaster, AL) dissolved in 2 mM β-mercaptoethanol. After 20 min gently stirring, the insoluble materials were pelleted by centrifugation at 170,200× g for 60 min at 4°C. Aliquots of the clear supernatant were stored at −70°C.

Proteoliposome reconstitution was performed as previously described [21]–[23]. The lipid mixture (45 mg/ml) was sonicated in a bath-type sonicator (Laboratory Supplies Inc, Hicksville, NY) at 18–23°C until the solution appeared clear, as described previously [22]. The detergent solubilized microsomal membrane extracts (500 to 700 µg) were mixed with the sonicated lipid mixture at a ratio of 1∶10 protein∶lipid (w/w) to a final volume of ∼1 ml and the reaction brought to a final volume of 4 ml by the addition of octylglucoside dilution buffer (100 mM KPO_4_, pH 7.5, 1.25% octylglucoside, 2 mM DTT, and 1% aprotinin). After mixing, the suspension was placed on ice for 20 min before being placed inside a dialysis cassette (PIERCE, Rockford, IL). For P_i_-loaded proteoliposomes, dialysis was performed overnight at 4°C against a phosphate buffer (20 or 50 mM KH_2_PO_4_, pH 7.0, 1 mM DTT, and protease inhibitors). For MOPS-loaded proteoliposones, dialysis was performed overnight at 4°C against a MOPS/K buffer (20 mM MOPS, pH 7.5 adjusted with KOH; 75 mM K_2_SO_4_; 2.5 mM MgSO_4_, 1 mM DTT, and protease inhibitors). The resulting proteoliposomes were pelleted by centrifugation at 170,200× g for 60 min 4°C, resuspended in 200 µl MOPS/K buffer, and used in transport assays. Proteoliposomes prepared from detergent solubilized microsomal membrane extracts of mock-infected cells were used as negative controls. The protein content in microsomes, detergent extract, and proteoliposomes was quantified by the Amido Black B protein estimation method as described previously [24].

### Western-blot analysis

For Western-blot analysis, proteins were resolved by electrophoresis through a 10% polyacrylamide-SDS gel and trans-blotted onto polyvinylidene fluoride membranes (Millipore). The membranes were incubated overnight with a rabbit polyclonal antibody against G6PT [12] or a mouse monoclonal antibody against the Flag epitope (Sigma-Alorich, St. Louis, MO). The membranes were then incubated with the appropriate horseradish peroxidase-conjugated second antibody and the immunocomplex visualized using the Immobilon western chemiluminescent HRP substrate (Millipore).

### Proteoliposomal transport assays

Proteoliposomal transport assays were performed at room temperature for various lengths of time using reaction mixtures containing 20 mM MOPS/K buffer, 25 µg/ml proteoliposomes, and either 0.1 mM [U-^14^C]G6P (50 µCi/µmol) or 0.5 mM ^32^P_i_. The ^32^P_i_ was prepared by boiling carrier-free ^32^P_i_ (MP Biochemical, Irvine, CA) in 1 ml of 1 N HCl for 3 h and diluting with an equal volume of 2 M K_2_HPO_4_ as described previously [25]. At each of the assay time points, 100 µl aliquots were withdrawn, filtered immediately through presoaked 0.22 µm nitrocellulose filters (Millipore), and washed with 20 mM MOPS/K buffer. The dried filters were then counted in a liquid scintillation counter. Inhibition of G6P or P_i_ transport by CHA (Sigma-Alorich) [7] or vanadate [26] was examined in reaction mixtures containing 20 mM MOPS/K buffer 25 µg/ml of 50 mM P_i_,-loaded G6PT-, SLC37A1- or SLC37A2-proteoliposomes, 0.1 mM [U-^14^C]G6P or 0.5 mM ^32^P_i_, and the respective inhibitor. After incubation at room temperature for 3 min, the reaction mixture was filtered and counted as described above.

### Real time PCR analysis

Total RNAs were isolated using TRIzol Reagent (Invitrogen). The mRNA expression was quantified by real-time RT-PCR, in triplicate, in an Applied Biosystems 7300 Real-Time PCR System using the gene-specific TaqMan® Gene Expression Assays and then normalized to β-actin RNA. The Applied Biosystems probes were: G6PT, Mm00484574_m1; SLC37A1, Mm00461949_m1; SLC37A2, Mm00451435_m1, SLC37A3, Mm00551183_m1 and β-actin, Mm00607939_s1.

### Statistical analysis

The unpaired t test was performed using the GraphPad Prism Program, version 4 (GraphPad Software, San Diego, CA). Values were considered statistically significant at *p*<0.05.

## Results

### The SLC37 members are ER-associated proteins

**Figure 1:**
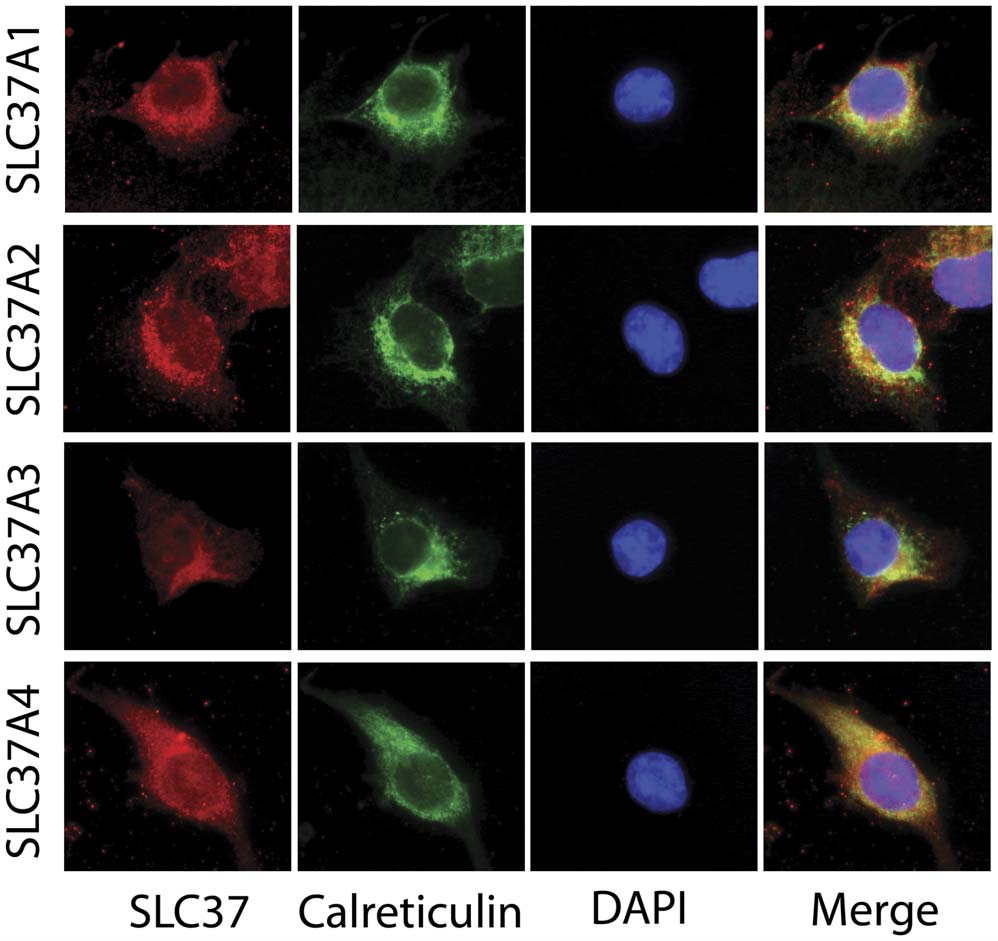
The SLC37 proteins are localized in the ER. Double immunofluorescence staining for SLC37A1, SLC37A2, SLC37A3, or G6PT (red fluorescence), endogenous calreticulin (green fluorescence), and DAPI in COS-1 cells infected with Ad-SLC37A1-5Flag, Ad-SLC37A2-5Flag, Ad-SLC37A3-5Flag, or Ad-G6PT-5Flag as described under Materials and Methods. Co-localization of SLC37 proteins with the ER-marker calreticulin is indicated by yellow in the merged image.

To determine the cellular localization of SLC37A2 and SLC37A3, COS-1 cells were infected with either Ad-SLC37A2-5Flag or Ad-SLC37A3-5Flag and visualized by double immunostaining for the Flag-tag and the ER-marker protein calreticulin [27]. Ad-SLC37A1-5Flag and Ad-G6PT-5Flag were used as positive controls. SLC37A2 and SLC37A3, co-localized with calreticulin (Figure 1) in the same manner as the positive controls, demonstrating their association with the ER.

### SLC37A1 and SLC37A2 but not SLC37A3 are P_i_-linked G6P antiporters

**Figure 2:**
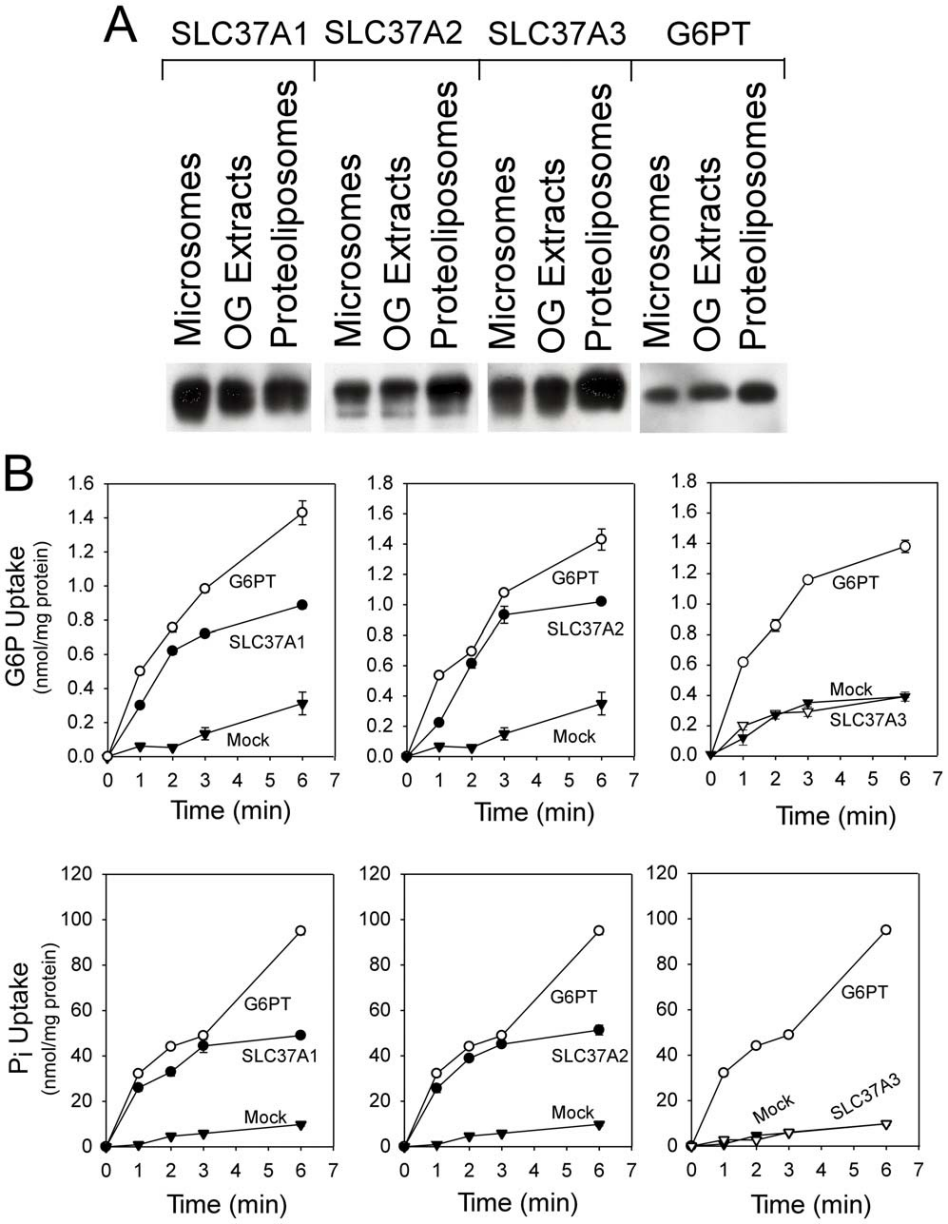
G6P and P_i_ uptake in SLC37 proteoliposomes. Microsomes were isolated from COS-1 cells infected with Ad-SLC37A1-5Flag, Ad-SLC37A2-5Flag, Ad-SLC37A3-5Flag or Ad-G6PT and proteoliposomes were reconstituted from the respective detergent solubilized microsomal membrane extracts as described under Materials and Methods. Results shown are from three independent experiments, each point determined in triplicate. (A) Western-blot analysis of SLC37 proteins during reconstitution of proteoliposomes from the respective microsomal membranes using a mouse monoclonal antibody against the Flag epitope or a rabbit polyclonal antibody against G6PT [12]. Each lane contains 10 µg protein. (B) G6P or P_i_ uptake in 50 mM P_i_ -loaded SLC37A1-, SLC37A2-, SLC37A3-proteoliposomes. G6P or P_i_ uptake in 50 mM P_i_ -loaded G6PT- and mock-proteoliposomes were used as controls.

To examine the G6P transport activity of SLC37A1, SLC37A2 and SLC37A3, proteoliposomes were reconstituted from detergent solubilized microsomal membrane extracts isolated from COS-1 cells infected with Ad-SLC37A1-5Flag, Ad-SLC37A2-5Flag, or Ad-SLC37A3-5Flag. Proteoliposomes reconstituted from mock- or Ad-G6PT-infected COS-1 cells were used as negative and positive controls, respectively. Microsome solubilization and proteoliposome reconstitution were monitored by Western blot analysis using an antibody against the Flag tag or G6PT [12] (Figure 2A).

The G6P uptake activity was measured in SLC37A1-, SLC37A2-, or SLC37A3-proteoliposomes loaded with 50 mM P_i_. The P_i_-loaded G6PT and mock proteoliposomes were used as positive and negative controls, respectively. As shown in an earlier study [3], labeled G6P was taken up efficiently into G6PT-proteoliposomes but not into mock-proteoliposomes (Figure 2B). G6P was also taken up efficiently in SLC37A1-proteoliposomes and SLC37A2-proteoliposomes (Figure 2B). In contrast, G6P transport activity in SLC37A3-proteoliposomes was indistinguishable from the activity of the mock-proteoliposomes (Figure 2B).

For P_i_ uptake assays, proteoliposomes were loaded with 50 mM P_i_. Again, labeled P_i_ was taken up efficiently into SLC37A1- and SLC37A2 and the control G6PT-proteoliposomes (Figure 2B). P_i_ uptake activity into SLC37A3-proteoliposomes was indistinguishable from the mock-proteoliposomes.

**Figure 3:**
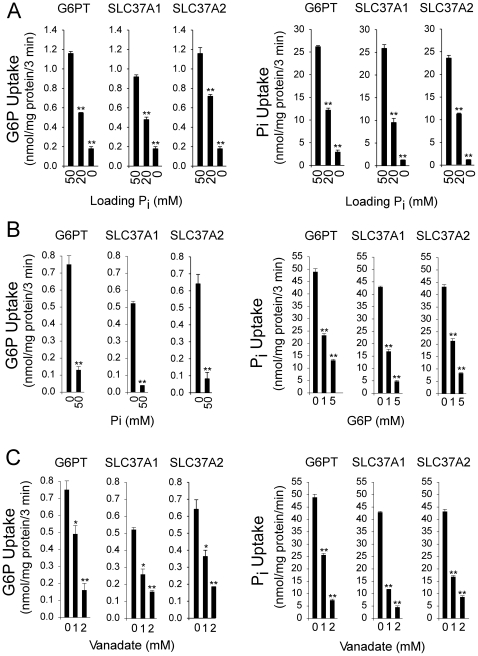
Effects of P_i_ loading, external G6P, P_i_ and vanadate on antiporter activity of SLC37 members. Proteoliposomes were reconstituted from detergent solubilized microsomal membrane extracts isolated from Ad-SLC37A1-5Flag, Ad-SLC37A2-5Flag, or Ad-G6PT infected COS-1 cells as described under Materials and Methods. Results shown are from three independent experiments, each point determined in triplicate. (A) Effects of P_i_ -loading on G6P or P_i_ uptake activity of SLC37 proteoliposomes. The concentrations of P_i_ loaded were 50 mM, 20 mM, and 0 mM (MOPS). (B) Inhibition of G6P uptake in 50 mM P_i_-loaded SLC37 proteoliposomes by external P_i_ or inhibition of P_i_ uptake in 50 mM P_i_-loaded SLC37 proteoliposomes by external G6P. (C) Effects of vanadate on G6P and P_i_ uptake activities of SLC37 proteoliposomes. Data are presented as the mean ± SEM. *p<0.05; **p<0.005.

Consistent with the findings for G6PT [3], the rates of G6P or Pi uptake correlated with the concentrations of P_i_ loaded in SLC37A1- or SLC37A2-proteoliposomes (Figure 3A). The antiporter activity was then examined in 50 mM P_i_-loaded SLC37A1- or SLC37A2-proteoliposomes by external G6P or P_i_. As expected, G6P uptake was marked inhibited by the addition of external P_i_ and P_i_ uptake was markedly inhibited by the addition of external G6P (Figure 3B).

To further elucidate antiporter activity of SLC37A1 and SLC37A2, we examined the effects of vanadate, a close structural and chemical mimic of P_i_ [26]. Vanadate inhibits G6P transport in hepatic microsomes [8] and P_i_-loaded G6PT-proteoliposomes [3]. The G6P and Pi uptake activity in P_i_-loaded SLC37A1- or SLC37A2-proteoliposomes was also sensitive to inhibition by vanadate (Figure 3C).

### Relative expression levels of SLC37 members in the liver, kidney, intestine, and pancreas

The liver and kidney play key roles in G6P metabolism and glucose homeostasis therefore any G6P transporters implicated in blood glucose homeostasis must be expressed in these organs. The intestine and pancreas also play roles in the control of glucose homeostasis [28], [29] and might similarly be expected to express the G6P transporters. We therefore examined the relative mRNA levels of the SLC37 members in the liver, kidney, intestine and pancreas. Quantitative RT-PCR analysis showed that in the liver, kidney, and intestine, G6PT transcripts were present at the highest levels and SLC37A2 at the lowest levels (Table 1). In the kidney, SLC37A3, which lacks G6P antiporter activity was expressed second to G6PT and in the intestine both SLC37A1 and SLC37A3 were expressed at significantly high levels (Table 1). In the pancreas, SLC37A3 transcripts were present at the highest levels, followed by G6PT, SLC37A1, and SLC37A2 (Table 1).

**Table 1:** The mRNA levels of SLC37 members, relative to G6PT/SLC37A4, in mouse liver, kidney, intestine, and pancreas.

SLC37 members	Liver	Kidney	Intestine	Pancreas
	*%*	*%*	*%*	*%*
G6PT/SLC37A4	100	100	100	100
SLC37A1	0.9±0.2	1.6±0.2	59.8±5.8	68.8±11.4
SLC37A2	1.3±0.2	1.0±0.2	4.4±2.8	3.8±0.4
SLC37A3	5.7±0.5	23.6±3.7	47.5±5.8	392.9±6.2

The expression levels of the SLC37A1, SLC37A2, SLC37A3, and G6PT/SLC37A4 transcripts were normalized to β-actin RNA and then scaled, for each tissue, relative to the SLC37A4 transcript which was arbitrarily assigned as 100%. Results reflect the average of three independent measurements of each transcript and are expressed as mean ± SEM.

### The antiporter activity of SLC37A1 and SLC37A2 is insensitive to inhibition by CHA

**Figure 4:**
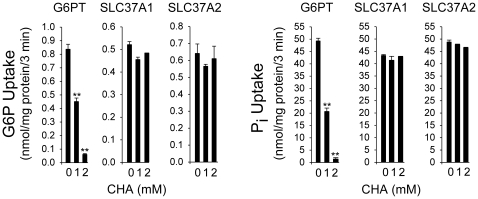
Effects of CHA on antiporter activity of SLC37A1 and SLC37A2. Proteoliposomes were reconstituted from detergent solubilized microsomal membrane extracts expressing SLC37A1, or SLC37A2 and were loaded with 50 mM P_i_ as described under Materials and Methods. The 50 mM P_i_-loaded G6PT proteoliposomes were used as a control. Results shown are from three independent experiments, each point determined in triplicate. Data are presented as the mean ± SEM. **p<0.005.

We then examined the effects of CHA, a specific inhibitor of G6P transport in the liver and kidney [7], on the antiporter activity of SLC37A1 and SLC37A2. As shown in earlier studies, G6P and P_i_ uptake in P_i_-loaded G6PT-proteoliposomes was markedly inhibited by CHA (Figure 4). Interestingly, this inhibitor had no effects on G6P or P_i_ uptake in P_i_-loaded SLC37A1- or SLC37A2-proteoliposomes (Figure 4).

### SLC37A1 and SLC37A2 cannot functionally couple with G6Pase-α or G6Pase-β

**Figure 5:**
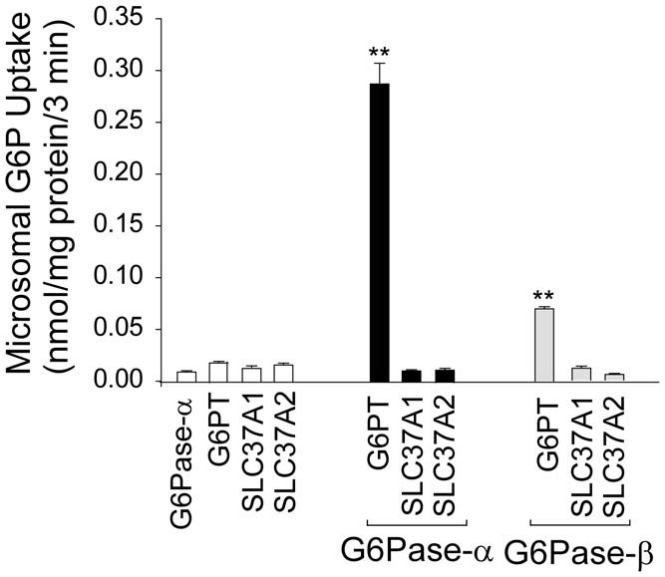
Effects of G6Pase-α and G6Pase-β on microsomal G6P transport activity of SLC37A1, SLC37A2 and G6PT. Microsomal membranes were isolated from COS-1 cells infected with Ad-G6Pase-α, Ad-G6PT, Ad-SLC37A1-5Flag, or Ad-SLC37A2-5Flag, co-infected with Ad-G6PT/Ad-G6Pase-α, Ad-SLC37A1-5Flag/Ad-G6Pase-α, or Ad-SLC37A2-5Flag/Ad-G6Pase-α, or co-infected with Ad-G6PT/Ad-G6Pase-β, Ad-SLC37A1-5Flag/Ad-G6Pase-β, or Ad-SLC37A2-5Flag/Ad-G6Pase-β as described in Materials and Methods. Results represent three independent experiments, with each data point determined in triplicate. Data are presented as the mean ± SEM. **p<0.005.

To examine coupling between the SLC37 transporters and G6Pase-α or G6Pase-β, we compared G6P uptake activity of microsomes expressing SLC37A1 or SLC37A2 in the presence or absence of G6Pase-α or G6Pase-β. Microsomes expressing SLC37A1, SLC37A2, or G6PT exhibited low levels of G6P transport activity (Figure 5). When co-expressed with G6Pase-α or G6Pase-β, both SLC37A1 and SLC37A2 showed little increase in G6P uptake activity. In contrast, the positive control microsomes co-expressing G6PT with G6Pase-α or co-expressing G6PT with G6Pase-β exhibited a markedly increased G6P uptake activity (Figure 5). As was shown in earlier studies [20], G6P uptake activity in microsomes co-expressing G6PT/G6Pase-α was 4.7-fold higher than the activity in microsomes co-expressing G6PT/G6Pase-β (Figure 5).

## Discussion

The solute-carrier (SLC) gene superfamily of membrane-bound transporters are organized into 55 gene families based largely on protein sequence homology and predicted substrate specificity [30]. The SLC37 family consists of 4 members, SLC37A1 through SLC37A4, named either organo-phosphate:P_i_ or sugar-phosphate:P_i_ exchangers [15]. While human SLC37A1 was originally isolated based on its similarity to *E. coli* glycerol-3-phosphate transporter, it was not found to be mutated in patients with glycerol kinase deficiency [13], suggesting glycerol-3-phosphate may not be its primary substrate. Despite this, there have been no functional assays to determine the substrate activity of SLC37A1. In contrast, SLC37A4, which was isolated independently both by homology to sequences of bacterial phosphate ester transporters [31] and by linkage analysis in GSD-Ib patients [32], has been extensively characterized genetically and functionally. The role of SLC37A4, more commonly called G6PT, is as a P_i_-linked G6P antiporter [3] that transports the G6P substrate from the cytoplasm into the ER lumen where the enzyme G6Pase-α hydrolyzes it to glucose and P_i_. The P_i_ released is in turn transported out of the ER lumen into the cytoplasm by the SLC37A4/G6PT antiporter activity [3]. In GSD-Ia and GSD-Ib patients, failure to hydrolyze G6P into glucose in the liver and kidney results in a loss of interprandial blood glucose homeostasis [5], [6]. Studies of hepatic microsomal G6P uptake from GSD-Ia mice have demonstrated that G6P uptake activity depends not only on G6PT, but also a functional coupling with G6Pase-α [8]. Microsomal G6P uptake activity is low in the absence of a functional G6Pase-α [8] but is greatly increased when a functional G6Pase-α is restored [9]. Studies have shown that there is no stringent genotype-phenotype correlation in GSD-Ib patients [33], suggesting the existence of a genetic modifier, probably another G6P transporter. Supporting this, Leuzzi et al. [18] showed additional G6P transport activities do exist in human cell lines. Therefore the SLC37 family proteins are natural candidates for the modifier/transporter, which may couple with G6Pase-α to compensate for loss of G6PT to maintain blood glucose homeostasis.

We set out to investigate if SLC37A1, SLC37A2 and/or SLC37A3 are G6P transporters and if so whether they are capable of coupling with G6Pase-α to impact blood glucose homeostasis. Since glycogenolysis and gluconeogenesis, the predominant sources of G6P in the cell, are cytoplasmic pathways, any candidate transporter must be an ER associated membrane protein, able to transport G6P into the lumen of the ER for the G6Pase-α activity. SLC37A4/G6PT [1], [2] and SLC37A1 [14] have been previously demonstrated to be embedded in the ER. We now demonstrated that SLC37A2 and SLC37A3 are similarly localized. Moreover the accessibility of their N-terminal Flag motif in permeabilized cells to the anti-Flag antibody also demonstrates that like SLC37A4 their N-terminus is on the cytoplasmic side of the ER membrane. Consistent with a previous demonstration that SLC37A4 has 10 transmembrane domains [1], [2], the protein sequences of the other SLC37 family members are also predicted to have between 10 and 12 domains. When assayed for G6P transport function, SLC37A1 and SLC37A2 both exhibited an activity similar to SLC37A4/G6PT, but SLC37A3 failed to show an uptake activity. Therefore, despite its similarity to glycerol-3-phosphate transporter, SLC37A1 is a G6P transporter. Of the three G6P transporters, all are capable of both heterologous G6P:P_i_ and homologous P_i_:P_i_ exchanges characteristic of SLC37A4/G6PT. While these are consistent with the SLC37A1 and SLC37A2 being possible modifiers of SLC37A4/G6PT, neither protein was capable of coupling with G6Pase-α. Moreover, while G6P transport activity responsible for blood glucose homeostasis is known to be sensitive to CHA inhibition [7], neither SLC37A1 nor SLC37A2 showed this sensitivity. Taken together, our data suggest that SLC37A1 and SLC37A2 are not implicated in blood glucose homeostasis.

This conclusion was further underlined by the expression profiles of the proteins. Interprandial blood glucose homeostasis is maintained predominantly by the release of glucose from G6P produced in the terminal steps of gluconeogenesis and glycogenolysis in the liver and kidney [5], [6], although intestine and pancreas also play roles in glucose homeostasis [28], [29]. While SLC37A3 is expressed primarily in the kidney, liver, and intestine, and is the second or third most abundant SLC37 transcript expressed in those organs, after SLC37A4/G6PT, it is the member that lacks G6P transport activity. SLC37A3 is also the most abundant SLC37 transcript in the pancreas. Given the sequence similarities, it is reasonable to expect SLC37A3 will have a sugar transport activity of some kind, but the exact functional role of SLC37A3 remains unknown. For SLC37A1 we also observe expression in the liver, kidney, intestine, and pancreas although the transcripts in the liver and kidney constitute less than 2% of the level seen for SLC37A4/G6PT. SLC37A1 is reported to be up-regulated by epidermal growth factor in breast cancer cells, leading to the suggestion that its biological role might be involved in phospholipid biosynthesis [14]. For SLC37A2, expression has been reported to be restricted to macrophages, spleen, and thymus [15], [17]. We find that SLC37A2 is also expressed in liver, kidney, intestine, and pancreas but at levels less than 5% of those of SLC37A4/G6PT. The report that SLC37A2 transcription is markedly increased upon macrophage differentiation [17] may point to a biological role with G6P in those cells. Together these results suggest that only SLC37A4 is implicated in blood glucose homeostasis and that the roles of the other SLC37 proteins lie in cells outside the gluconeogenic tissues. It will be of interest in the field to understand the physiological role of the other SLC37 proteins and determine if mutations underlie metabolic disorders other than GSD-I.

In summary, we have shown that of the four members in the SLC37 family, SLC37A1 and SLC37A2 exhibit a P_i_-linked G6P antiporter activity similar to that of the well characterized SLC37A4/G6PT. However, their inability to couple with G6Pase-α or G6Pase-β combined with their lack of sensitivity to CHA, and low levels of expression in the gluconeogenic organs suggests that their biological roles lie outside of blood glucose homeostasis.
